# Flow Characteristics of Barium Thickened Liquids during Swallowing in Healthy Adults

**DOI:** 10.1007/s00455-026-10937-4

**Published:** 2026-02-21

**Authors:** Pimchanok Tuakta, Akapong Kongjaroen, Pawadee Methacanon, Chaiwut Gamonpilas, Nilnetre Mahathanaruk, Sith Phongkitkarun, Paitoon Benjapornlert

**Affiliations:** 1 Department of Rehabilitation Medicine, Faculty of Medicine Ramathibodi Hospital, Mahidol University, 270 Rama VI Rd, Thung Phaya Thai, Ratchathewi, Bangkok, 10400 Thailand; 2 Advanced Polymer Technology Research Group, National Metal and Materials Technology Center (MTEC), NSTDA, 111 Thailand Science Park, Phahonyothin Road, Khlong Nueng, Khlong Luang, Pathumthani, 12120 Thailand; 3 Department of Otorhinolaryngology, Faculty of Medicine Ramathibodi Hospital, Mahidol University, 270 Rama VI Rd, Thung Phaya Thai, Ratchathewi, Bangkok, 10400 Thailand; 4 Department of Diagnostic and Therapeutic Radiology, Faculty of Medicine Ramathibodi Hospital, Mahidol University, 270 Rama VI Rd, Thung Phaya Thai, Ratchathewi, Bangkok, 10400 Thailand

**Keywords:** Deglutition, Rheology, International dysphagia diet standardisation initiative (IDDSI), Videofluoroscopic swallowing study (VFSS), Healthy adult

## Abstract

Modification of liquid viscosity complying with the International Dysphagia Diet Standardisation Initiative (IDDSI) framework is crucial in managing swallowing difficulties in individuals with dysphagia. Despite having similar viscosities, different thickening agents may exhibit varied extensional properties, potentially influencing bolus formation and the movement of oropharyngeal and hyolaryngeal structures during swallowing. This study aims to evaluate the effects of various thickening agents on swallowing behaviour and the extensional properties of boluses across IDDSI levels 1 to 4, using the Videofluoroscopic Swallowing Study (VFSS). Thirty healthy participants consumed 10 mL of liquids thickened with five different thickening agents, prepared at each IDDSI level 1 to 4, during VFSS. The bolus head during swallowing onset, the movement of oropharyngeal and laryngeal structures and the bolus aspect ratio (BAR) were analysed across different thickening agents. With increasing IDDSI levels, the bolus was observed at progressively higher anatomical levels—residing in the valleculae or hypopharynx at IDDSI level 1, and in the oral cavity at IDDSI level 3 and IDDSI level 4. Laryngeal closure duration (LCD) significantly decreased with increasing IDDSI levels (from 1 to 3 and 4). In addition, ThickenUp^®^ Clear (TUC) yielded the highest LCD among all thickening agents. The BAR remained stable across IDDSI levels and thickening agents, with no significant differences noted. In healthy adults, thicker consistencies positioned the bolus higher in the oropharynx and prolonged laryngeal closure, supporting safer swallowing. Gum-based thickeners produced more cohesive and stable boluses than starch-based agents, allowing more controlled bolus flow and airway protection.

**Supplementary Information:**

The online version contains supplementary material available at 10.1007/s00455-026-10937-4.

## Introduction

Swallowing disorders, or dysphagia, are prevalent among individuals with neurological diseases, head and neck cancers, and older adults. Dysphagia affects approximately 8% of the population across the lifespan, contributing to malnutrition, dehydration, choking, suffocation, and aspiration [1]. In severe cases, aspiration can result in pneumonia, a leading cause of mortality worldwide [2, 3]. Patients with dysphagia often experience difficulty in chewing, bolus formation, oral transportation, and clearing liquid and food residues from the oropharyngeal cavity [4].

Modification of liquid viscosity and food texture remains a fundamental strategy of dysphagia management, aiming to reduce aspiration risk and improve swallowing safety. Thickened liquids slow bolus flow and reduce its spread in the pharyngeal cavity, providing additional time for airway closure, while texture modified foods facilitate mastication and cohesive bolus formation, thereby reducing pharyngeal residue in the valleculae and pyriform sinus [5–7].

Historically, the National Dysphagia Diet (NDD) classified thickened liquids into four categories based on shear viscosity () measured at a defined shear rate of 50 s^−1^: thin liquid ( < 0.051 Pa.s), nectar-thick liquid ( = 0.051–0.350 Pa.s), honey-thick ( = 0.351–1.750 Pa.s), and spoon-thick ( > 1.750 Pa.s) [3, 5, 8]. More recently, the International Dysphagia Diet Standardisation Initiative (IDDSI) has introduced a globally applicable framework that categorises drinks into five levels: thin (IDDSI level 0), slightly thick (IDDSI level 1), mildly thick (IDDSI level 2), moderately thick (IDDSI level 3), and extremely thick (IDDSI level 4) [9, 10]. A syringe flow test is typically used to reliably classify liquids at IDDSI Levels 0 to 3. Furthermore, the fork drip test is recommended for assessing IDDSI Levels 3 and 4.

Thickening agents – typically gum-based, starch-based or blends – are widely used to achieve targeted IDDSI levels [10]. These thickening agents can significantly influence the bolus’s flow behaviour as it transits from the oral cavity into the pharyngeal cavity during swallowing [11, 12]. In addition, starch-based thickeners commonly exhibit time-dependent viscosity changes due to their nature as a solid suspension, while gum-based thickeners remain comparatively more stable [13]. Beyond shear viscosity, which governs resistance to steady flow, the cohesiveness of a bolus is equally important for swallowing safety. Cohesiveness is closely related to extensional rheology, which describes a fluid’s resistance to stretching and helps prevent bolus fragmentation during the inherent elongational flows of bolus swallowing [14]. An ideal swallow-safe bolus has been described as moist, cohesive, and slippery [15].

Numerous studies have examined the viscoelastic and extensional rheological properties of thickened liquids in relation to swallowing safety [12, 14, 16]. Videofluoroscopic swallowing studies (VFSS) in healthy adults demonstrated that extensional viscosity plays a more decisive role than shear viscosity in determining bolus shape at the upper oesophageal sphincter [17]. However, the VFSS results using pharyngeal transit time and bolus aspect ratio limited the physiological depth of interpretation. Gum-based thickeners with higher maximum extensional viscosity formed shorter, more cohesive boluses with reduced pharyngeal coating, whereas starch-based thickeners matched in shear viscosity at a representative rate of 50 s⁻¹ but lower in extensional viscosity produced elongated, heterogeneous boluses susceptible to filament break-up [17]. Additionally, a range of liquids matched to equivalent shear viscosity under the NDD criterion have been shown to exhibit substantially different extensional responses, resulting in distinct in vivo bolus flow behaviours during swallowing [11, 12].

In clinical VFSS, barium sulfate suspensions are routinely used to provide radiopacity [18]. However, barium-containing thickened liquids can differ significantly from their non-barium counterparts in key physical and rheological parameters, including higher density, shear viscosity, and yield stress [19, 20]. These changes can influence bolus flow and residue; however, systematic in vivo studies examining how the type of thickening agent interacts with barium to affect swallowing dynamics are still limited.

Despite these insights on the rheological factors influencing swallowing, relatively few studies have systematically compared different thickening agents using VFSS. In particular, data linking the in-situ flow properties of barium-thickened liquids with VFSS-derived swallowing measures remains scarce. This study, therefore, examines the effects of various thickening agents on swallowing function in healthy adults, using VFSS to characterise bolus flow, oropharyngeal and hyolaryngeal kinematics and the in-situ rheological properties of the bolus during swallowing.

## Methodology

### Participants

Thirty healthy adults (mean age 40.7 ± 10.7 years; BMI 26.6 ± 6.3 kg/m²) were enrolled, including 23 females and seven males. No significant differences in age or BMI were observed between sexes (*p* = 0.16 and *p* = 0.74, respectively). Participants were required to have no history of swallowing problems or neurological disorders that could affect their swallowing function prior to the study. Additionally, participants with a risk of dysphagia, indicated by a score of more than three on the Eating Assessment Tool (EAT-10) [21], were excluded. Informed consent was obtained from all participants, and the study was approved by the Ethics Committee of the Faculty of Medicine, Ramathibodi Hospital, Mahidol University (Approval No. MURA2021/944).

###  Sample Preparation

The study used five commercially available thickening agents, each with a different main compound: (1) Resource Thicken U3p Clear (TUC), a xanthan gum-based product by Nestle; (2) Quik Thik (QT), also xanthan gum-based, produced by Dr Macleod’s Medical Food; (3) Supercol (SP), which is guar gum-based and manufactured by Supercol; (4) Purathick (PT), a tara gum-based product from Parapharma Tech; and (5) Thicken Up (TU), a modified maize starch-based product by Nestle.

For barium stimulus preparation, 100 g of barium sulfate powder (Emprove Essential) was dispersed into 300 mL of mineral water to form the barium suspension used in the study. Thickened liquids were subsequently prepared to target IDDSI Levels 1–4, following the manufacturer’s preparation guidelines for each commercial thickening powder. The concentrations required for each thickener to reach the intended IDDSI levels are reported in Table 1. The IDDSI classification of each thickened barium liquid was verified using an IDDSI-aligned flow tester to minimise operator bias and ensure consistency accuracy [22]. While barium addition altered the effective shear viscosity relative to matched, non-barium thickened liquids (*Figure *S1,* Supplementary*), all barium-thickened samples were validated to fall within their assigned IDDSI levels.

**Table 1:** Concentrations of each commercial thickening powder for different liquid consistencies based on IDDSI

Sample	IDDSI Level 1	IDDSI Level 2	IDDSI Level 3	IDDSI Level 4
TUC	0.80	1.90	3.50	11.20
QT	1.50	2.50	4.50	12.00
ST	0.36	0.56	0.77	1.40
PU	0.80	1.20	1.70	3.00
TU	1.50	2.20	3.40	5.20

### Rheological Measurement

Shear rheology was conducted using a stress-controlled rheometer (Kinexus ultra plus, Malvern Instrument, Malvern, UK) equipped with a parallel plate geometry (40 mm in diameter and a 1 mm gap size). A parallel plate geometry was used in this study to ensure consistency and to avoid particle-induced artefacts as the average particle size of modified maize (TU) sample D [3, 4] = 45.77 ± 0.83 μm as shown in *Figure S2 of the Supplementary data*) was comparable to the truncated gap of a cone and plate system. After sample loading, all samples were pre-sheared at 0.1 s^− 1^ for 60 s and equilibrated for 60 s at 25 °C to remove the loading history. Shear viscosity was then measured over the applied shear rates of 0.1–500 s^− 1^. Three replicates were used for each sample, and all tests were conducted at 25 °C [23, 24].

### IDDSI Measurements [10, 18]

IDDSI flow measurement was conducted using an in-house flow tester in accordance with the IDDSI testing framework [10]. The apparatus employed a 10 mL Luer slip tip syringe (Nipro Corporation Limited, Thailand) measuring 61.5 mm from the 0 mL to 10 mL line, with a taper angle of approximately 28˚ at the bottom of the barrel. A 10 mL liquid sample was loaded into the syringe and allowed to flow under gravity for 10 s, after which the remaining volume was recorded and used to determine the corresponding IDDSI level. While the syringe differed from the BD 303,134 model specified in the IDDSI framework, comparative tests using both types of syringes across liquids spanning IDDSI levels 1 to 4 revealed no significant differences in residual volume. The Nipro syringe was used in this study due to its ease of procurement.

In addition to the flow test, fork drip and spoon tilt tests were performed to assess the flow characteristics and classify samples according to IDDSI level 4 criteria. In the fork drip test, a sample was placed on a fork and visually examined for any dripping through its tines. In the spoon tilt test, a sample was placed on a spoon and gradually tilted until it detached. The amount of residue remaining on the spoon was subsequently inspected and photographed.

### VFSS Procedure

Participants sat upright (90°) during VFSS, recorded at 30 frames per second in a lateral projection view. The study began with one spoon of extremely thick liquid (IDDSI level 4), followed by 10 mL each of moderately thick (IDDSI level 3), mildly thick (IDDSI level 2), and slightly thick (IDDSI level 1) liquids. Each liquid was prepared using five different commercial thickening agents. The VFSS was conducted by a certified physiatrist and an occupational therapist specialising in dysphagia.

### Outcome Measurement

**Fig. 1:**
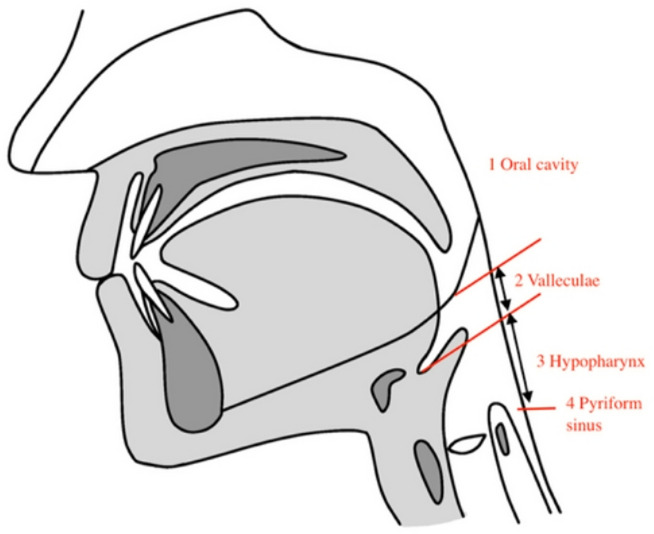
The edge of the bolus head at first swallowing response was demonstrated in four regions: (1) oral cavity (from the lip to before reaching the lower border of the mandible (LBM)), (2) valleculae (from LBM to base of valleculae), (3) hypopharynx (from the edge of the epiglottis to before reaching the floor of the pyriform sinus), and (4) pyriform sinus (at the pyriform sinus)

**Fig. 2:**
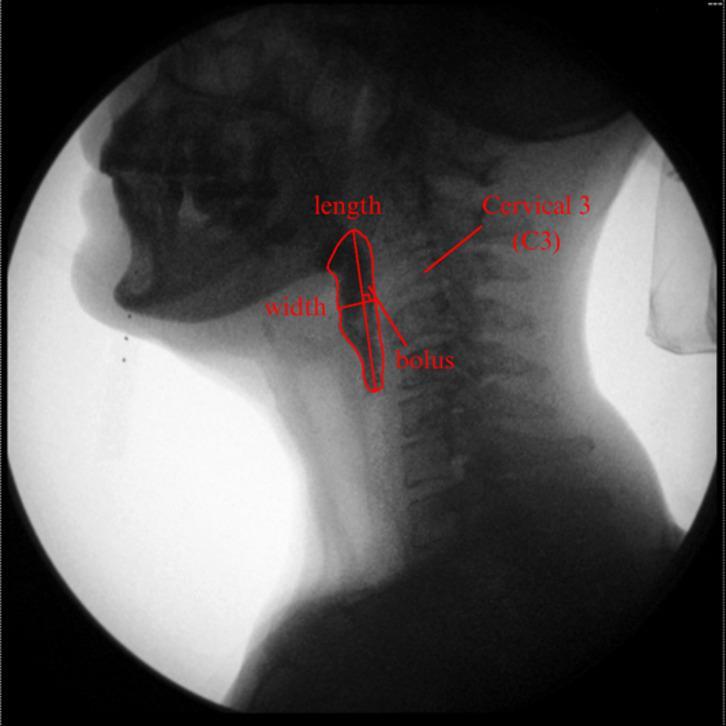
Bolus aspect ratio (BAR) at the upper oesophageal sphincter (UES) opening, where BAR is calculated as the ratio of bolus length to bolus width at the point of maximal UES opening during swallowing

At each swallow, the passage of bolus at the first swallow related to the movement of hyolaryngeal and pharyngeal complexes and upper oesophageal sphincter (UES) was analysed, as defined in Table 2 [25]. The edge of the bolus head at first swallowing response was demonstrated in four regions: (1) oral cavity (from the lip to before reaching the lower border of the mandible (LBM)), (2) valleculae (from LBM to base of valleculae), (3) hypopharynx (from the edge of the epiglottis to before reaching the floor of the pyriform sinus), and (4) pyriform sinus (at the pyriform sinus), as shown in Fig. 1 [26]. The aspect ratio of the bolus (BAR) at the UES opening [17] was described. It was the proportion of bolus length from head to tail and width at the C3 level at the time of UES opening, referred to as the bolus aspect ratio (BAR), as shown in Fig. 2. The ImageJ software (National Institutes of Health, Wayne Rusband) was used for analysing the BAR. Two independent researchers analysed the VFSS records; a difference of fewer than 0.1 s for the continuous data was allowable. The total agreement between the researchers was admitted for the ordinal data (edge of the bolus) and the ratio.

**Table 2:** The hyolaryngeal and pharyngeal complexes’ movement during bolus passage definition

Laryngeal closure duration (LCD)	The duration from the complete closure to the reopening of the laryngeal vestibule occurs at the arytenoids and the base of the epiglottis.
UES opening duration	The time from when the UES opens to allow bolus passage until it starts to close.
Vallecular aggregation time (VAT)	The time from when chewed food accumulates in the valleculae to when the leading edge of the bolus passes the epiglottis and enters the hypopharynx.
Upper hypopharynx transit time (UHT)	The time begins at the end of VAT and ends when the leading edge of the bolus reaches the floor of the pyriform sinus.
Lower hypopharynx transit time (LHT)	The time from the end of UHT until the tail of the bolus passes through the UES.
Stage transition duration (STD)	The time from when the hyoid bone begins maximum elevation until the bolus passes the ramus of the mandible.
Pharyngeal response duration (PRD)	The time from when the hyoid bone begins maximum elevation to when the hyoid bone returns to its resting position.
Pharyngeal transit duration (PTD)	The time from when the tail of the bolus enters the UES until the head of the bolus passes the ramus of the mandible.
Bolus aspect ratio (BAR)	The aspect ratio of the bolus at the UES opening is defined as the ratio of bolus length (from tail to head) to its width at Cervical 3 (C3) (length/width).

To compare the mean of each variable between the IDDSI levels 2 to 4 and IDDSI level 1, a t-test was used. Statistical significance was set at *P* < 0.05. Analysis was conducted using Stata version 18.

## Results

### Rheology of barium-thickened Liquids

**Fig. 3:**
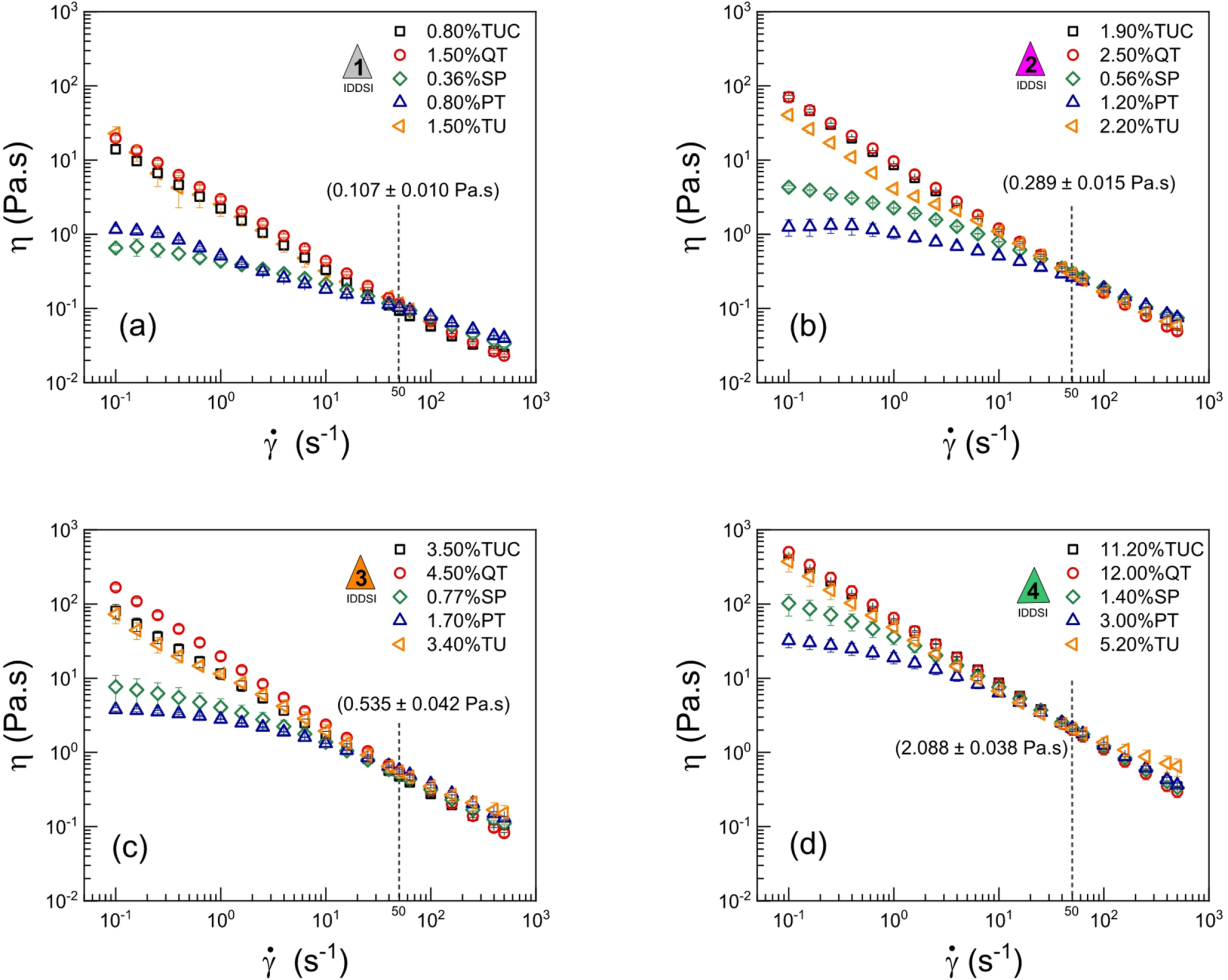
Shear viscosity as a function of shear rate for barium-thickened liquids prepared with different commercial thickening powders at concentrations corresponding to each IDDSI liquid consistency: (**a**) IDDSI level 1, (**b**) IDDSI level 2, (**c**) IDDSI level 3, and (**d**) IDDSI level 4

Rheological properties of barium-thickened liquids used for VFSS examination were examined for a range of commercial thickening powders and concentrations. For each level of IDDSI liquid consistency (IDDSI levels 1 to 4), initial experiments were conducted to determine the concentration of each thickening powder that yields a similar viscosity at a shear rate of 50 s^− 1^, which is typically a defined shear rate characteristic for swallowing [8, 12, 14]. The detailed experimental protocols and results underpinning this work have been comprehensively reported in our previous study [22], which provides the methodological foundation for the present investigation. The flow curves of all barium-thickened liquids are shown in Fig. 3*(a)-(d)* for IDDSI consistency levels 1 to 4, respectively. All samples exhibited non-Newtonian behaviour with shear-thinning characteristics. At the representative shear rate of 50 s^− 1^, the measured viscosities were consistent within each IDDSI liquid consistency level, with mean values of 0.107 ± 0.010, 0.289 ± 0.015, 0.535 ± 0.042, and 2.088 ± 0.038 Pa · s, for IDDSI levels 1 to 4, respectively.

In contrast, the low-shear viscosities, which are particularly relevant to product stability, differed markedly among samples. Based on these findings, PT- and SP-thickened liquids generally exhibited lower viscosities at low shear rates, suggesting reduced stability relative to the other formulations. These differences can be attributed to the varied active thickening compounds used in each thickening powder formulation. According to the product labels, PT and SP consist of tara gum and guar gum, respectively, as their primary thickening agents, while both TUC and QT consist of xanthan gum, and TU is formulated with modified starch. These findings are in good agreement with earlier studies on thickened liquids prepared using different active hydrocolloids [11, 17, 23, 24].

### IDDSI Testing

**Fig. 4:**
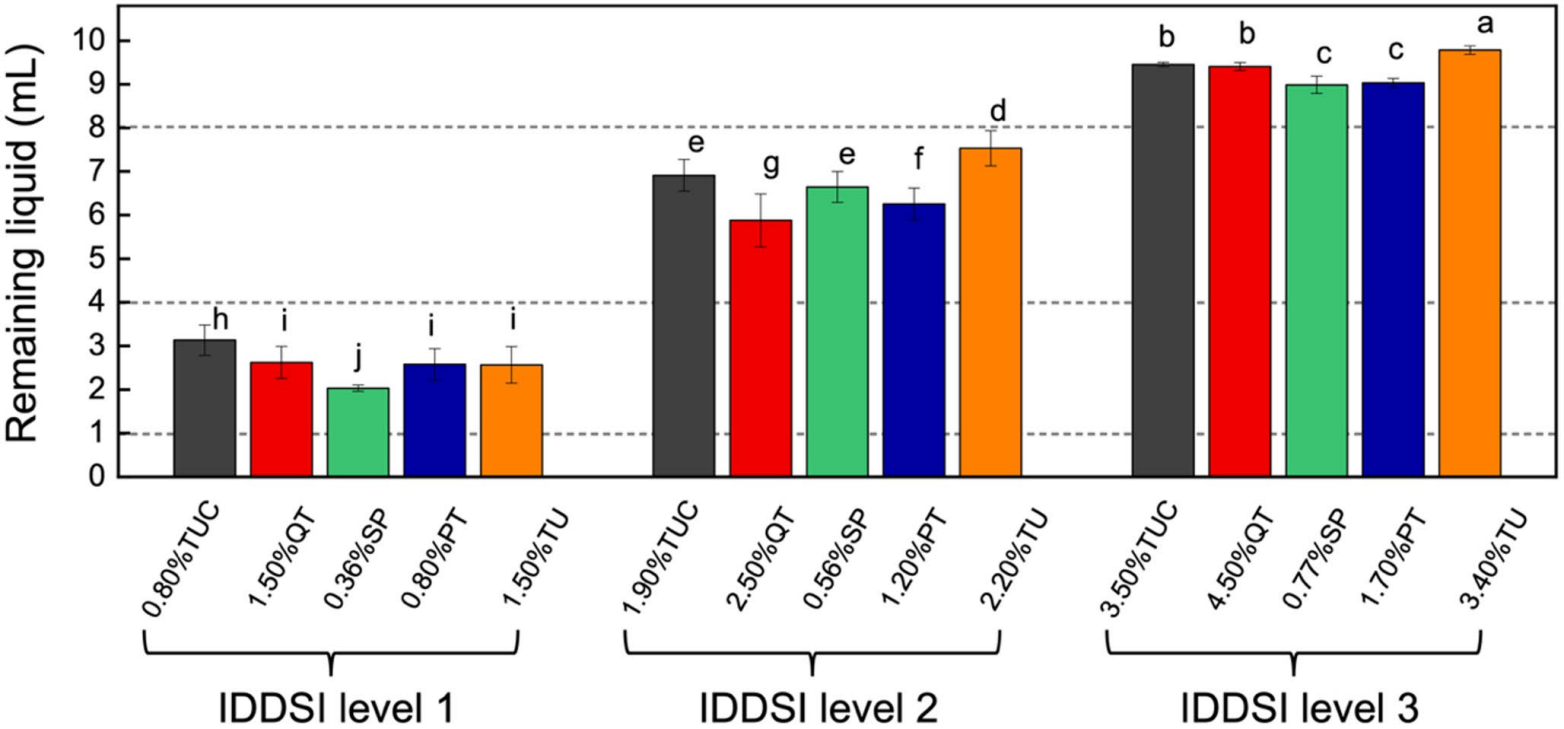
Liquid thickness of barium-thickened samples as determined by the IDDSI flow test. Different letters (a-i) indicate significant differences among the samples (*P* < 0.05)

The flow characteristics of each barium-thickened sample were evaluated using the IDDSI flow, fork drip, and spoon tilt test [27]. For samples complying with IDDSI levels 1–3, flow test results are presented in Fig. 4. Although viscosity at a shear rate of 50 s^− 1^ was adjusted to be similar for IDDSI level, the residue volume after the flow test varied slightly depending on the thickening powder. The residue liquid volumes were 2.59 ± 0.39 mL, 6.64 ± 0.63 mL, and 9.33 ± 0.32 mL for IDDSI levels 1, 2, and 3, respectively. IDDSI level 4 samples did not flow from the testing syringe; thus, their results are not included in the same figure.

**Fig. 5:**
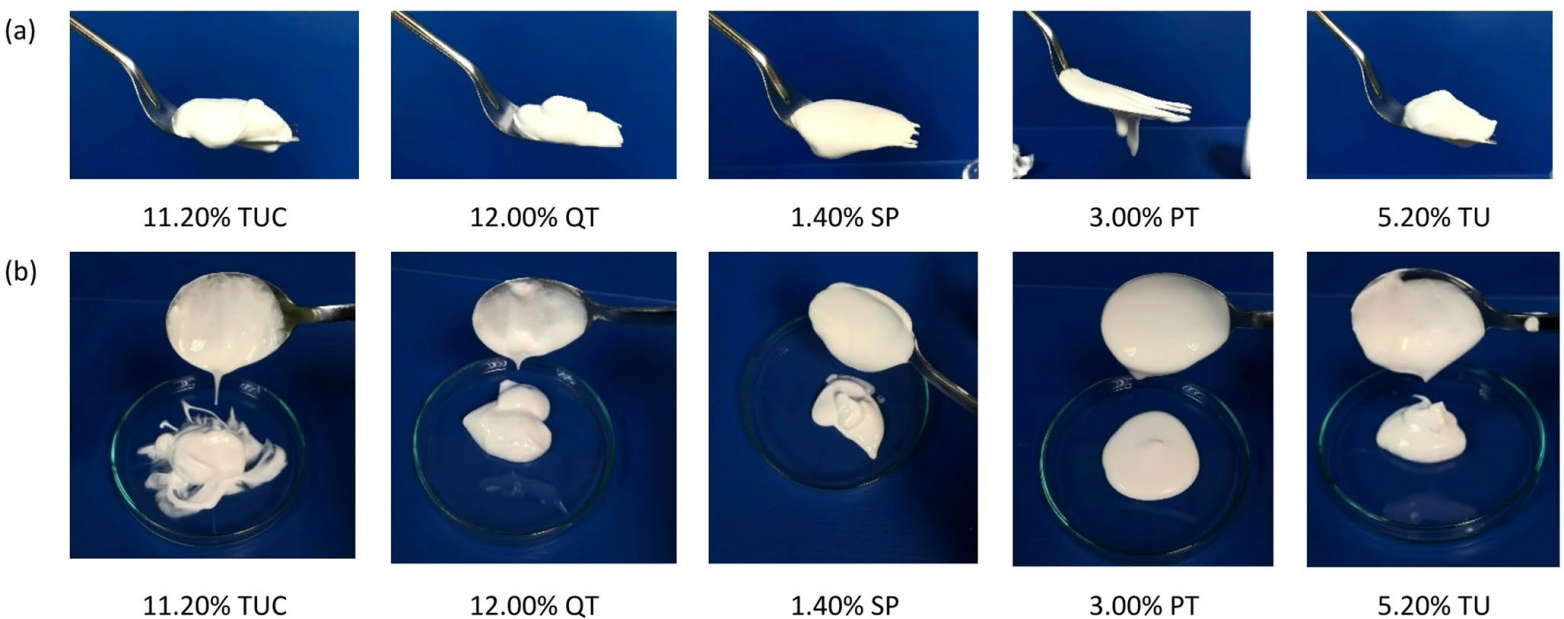
Flow characteristics of IDDSI level 4-compliant barium-thickened samples evaluated by (**a**) the fork drip test and (**b**) the spoon tilt test

The liquid thickness of IDDSI level 4 samples was further characterised using the fork drip and spoon tilt tests (Fig. 5). In the fork drip test, none of the samples flowed through the fork tines; however, the PT sample appeared less thick, forming a short tail beneath the fork. No continuous dripping was observed in any samples. In the spoon tilt experiments, all samples slid off the spoon upon tilting, leaving a visible residue on the spoon. All these samples were next used in the VFSS experiments.

### Videofluoroscopic Swallowing Study (VFSS)

#### The Edge of the Bolus during the Swallowing Onset

Table 3 presents the median bolus positions for all samples. VFSS analysis showed that the median bolus position tended to remain higher as the IDDSI level increased. At IDDSI level 1, most samples positioned the bolus in either the valleculae or hypopharynx, with TUC and TU located in the hypopharynx, while QT and SP in the valleculae. By contrast, at IDDSI levels 3 and 4, all samples retained the bolus within the oral cavity. Hyolaryngeal and pharyngeal movement phenomenon, the duration of the laryngeal closure, and the onset of UES opening during swallowing for different IDDSI levels are summarised in Table 4, while corresponding results for different thickening powders are presented in Table 5.

**Table 3:** The median bolus positions for each sample and IDDSI level

Sample	IDDSI level 1	IDDSI level 2	IDDSI level 3	IDDSI level 4
TUC	3	3	1	1
QT	2	2	1	1
SP	2,3	3	1	1
PT	2,3	3	3	1
TU	3	3	1	1

TUC = Resource Thicken Up Clear, QT = Quik Thik, SP = Supercol, PT = Purathick, TU = Thicken Up

1 = oral cavity, 2 = valleculae, 3 = hypopharynx, 4 = pyriform sinus

**Table 4:** Movement of hyolaryngeal and pharyngeal complexes at different IDDSI levels. Values are presented as mean ± SD

VFSS parameters	IDDSI level 1	IDDSI level 2	IDDSI level 3	IDDSI level 4
LCD (ms)	65.6 ± 4.24^a^	59.6 ± 3.32^a^	61.2 ± 3.91^a, b^	58.2 ± 5.31^a, b^
UHT (ms)	7.2 ± 0.84	7.4 ± 0.89	5.6 ± 1.08	6.6 ± 0.73
LHT (ms)	62.2 ± 2.39	60.0 ± 1.84	65.0 ± 2.73^a^	65.8 ± 3.55^a^
STD (ms)	-7.0 ± 2.45	-6.0 ± 2.00	-3.8 ± 2.77	-0.2 ± 2.77
PRD (ms)	48.8 ± 1.94	49.6 ± 2.13	52.2 ± 2.78	50.6 ± 3.24^a^
PTD (ms)	77.4 ± 2.14	74.2 ± 2.18	76.8 ± 3.00	80.4 ± 4.01
BAR	3.75 ± 0.18	3.79 ± 0.22	3.58 ± 0.25	3.57 ± 0.28

LCD = laryngeal closure duration, UHT = upper hypopharyngeal transit time, LHT = lower hypopharyngeal transit time, STD = stage transit duration, PRD = pharyngeal response duration, PTD = pharyngeal transit duration, BAR = bolus aspect ratio

^a^statistically significant difference (*P* < 0.05) when compared to the estimated IDDSI 0

^b^statistically significant difference (*P* < 0.05) when compared to IDDSI level 1

**Table 5:** The hyolaryngeal and pharyngeal complex’s movement in different thickening agents. Values are presented as mean ± SD

IDDSI level	Sample	Time (ms)						BAR
		**LCD**	**UHT**	**LHT**	**STD**	**PRD**	**PTD**	
1	TUC	72 ± 17^a^	6 ± 5	64 ± 13	-6 ± 6	50 ± 15	76 ± 16	3.60 ± 0.74
	QT	68 ± 16^ab^	7 ± 3	64 ± 11	-6 ± 10	49 ± 11	77 ± 15	3.99 ± 0.91
	SP	62 ± 13^b^	8 ± 4	62 ± 10	-6 ± 7	49 ± 11	76 ± 15	3.98 ± 1.15
	PT	60 ± 10^b^	7 ± 4	62 ± 13	-6 ± 8	50 ± 11	77 ± 17	3.64 ± 0.75
	TU	66 ± 17^ab^	8 ± 5	59 ± 8	-11 ± 20	47 ± 10	81 ± 20	3.52 ± 0.64
2	TUC	59 ± 10	7 ± 5	60 ± 11	-8 ± 13	52 ± 15	73 ± 16	3.69 ± 0.80
	QT	61 ± 14	7 ± 4	60 ± 11	-6 ± 8	50 ± 11	74 ± 17	3.91 ± 0.62
	SP	60 ± 11	8 ± 4	61 ± 12	-4 ± 8	49 ± 11	73 ± 16	3.78 ± 0.83
	PT	60 ± 13	7 ± 4	61 ± 11	-6 ± 12	51 ± 13	75 ± 14	3.94 ± 1.26
	TU	58 ± 16	7 ± 4	58 ± 17	-6 ± 9	46 ± 13	76 ± 29	3.62 ± 0.72
3	TUC	55 ± 8^c^	6 ± 3	64 ± 15	-4 ± 8	52 ± 13	74 ± 17	3.67 ± 0.80
	QT	66 ± 21^ab^	5 ± 3	63 ± 15	-3 ± 6	51 ± 12	75 ± 18	3.55 ± 0.68
	SP	61 ± 15^abc^	6 ± 3	65 ± 15	-4 ± 10	53 ± 12	78 ± 20	3.62 ± 0.86
	PT	67 ± 18^a^	9 ± 15	68 ± 17	-5 ± 11	52 ± 13	82 ± 23	3.52 ± 0.90
	TU	57 ± 9^bc^	5 ± 2	66 ± 18	-1 ± 6	53 ± 10	75 ± 18	3.56 ± 0.57
4	TUC	60 ± 14	7 ± 2	64 ± 20	1 ± 7	52 ± 11	86 ± 32	3.46 ± 0.68
	QT	59 ± 12	6 ± 3	63 ± 18	-1 ± 8	52 ± 6	79 ± 24	3.50 ± 0.59
	SP	56 ± 8	7 ± 3	65 ± 17	0 ± 6	50 ± 15	77 ± 20	3.65 ± 0.86
	PT	60 ± 10	6 ± 3	66 ± 22	0 ± 6	51 ± 12	80 ± 27	3.75 ± 0.89
	TU	59 ± 13	6 ± 2	67 ± 22	-1 ± 7	48 ± 15	80 ± 28	3.49 ± 0.50

TUC = Resource Thicken Up Clear, QT = Quik Thik, SP = Supercol, PT = Purathick, TU = Thicken Up

LCD = laryngeal closure duration, UHT = upper hypopharyngeal transit time, LHT = lower hypopharyngeal transit time, STD = stage transit duration, PRD = pharyngeal response duration, PTD = pharyngeal transit duration, BAR = bolus aspect ratio

Superscripts (a-c) indicate significant differences in the data in the same column for each IDDSI level (*P* < 0.05)

#### Laryngeal Closure Duration (LCD)

The overall mean LCD decreased from IDDSI level 1 (65.6 ± 4.24 ms) to IDDSI level 2 (59.6 ± 3.32 ms) and remained consistent at higher IDDSI levels. A significant reduction in LCD was observed for IDDSI levels 3 (61.2 ± 3.91 ms, *p* = 0.041) and 4 (58.2 ± 5.31 ms, *p* = 0.018) compared to IDDSI level 1. Among the studied thickening agents, TUC consistently demonstrated the highest LCD across all IDDSI levels, with a peak at IDDSI level 1 (72 ± 17 ms). PT had the lowest LCD at IDDSI levels 1 (60 ± 10 ms) and 2 (60 ± 13 ms). Significant differences were found between agents at IDDSI levels 1 and 3.

#### Upper Hypopharyngeal Transit time (UHT)

No significant changes in UHT were observed across IDDSI levels, with values ranging from 7.2 ± 0.84 ms at IDDSI level 1 to 6.6 ± 0.73 ms at IDDSI level 4. Similarly, no significant differences were found between thickening agents for UHT at any IDDSI level.

#### Lower Hypopharyngeal Transit time (LHT)

LHT remained relatively stable across IDDSI levels, with no significant differences observed. The values ranged from 60.0 ± 1.84 ms at IDDSI level 2 to 65.8 ± 3.55 ms at IDDSI level 4. Among the studied thickening powders, PT exhibited the highest LHT at IDDSI level 3 (68 ± 17 ms), but no significant differences were found related to the agent.

#### Stage Transition Duration (STD)

STD was consistently negative across all IDDSI levels, indicating a shortened stage transition. However, no significant differences were found between IDDSI levels or thickening agents. The values ranged from − 7.0 ± 2.45 ms at IDDSI level 1 to -0.2 ± 2.77 ms at IDDSI level 4.

#### Pharyngeal Response Duration (PRD)

PRD remained stable across IDDSI levels, with values ranging from 48.8 ± 1.94 ms at IDDSI level 1 to 52.2 ± 2.78 ms at IDDSI level 3, with no significant differences between levels. Similarly, no agent-related differences were observed for PRD at any IDDSI level.

#### Pharyngeal Transit Duration (PTD)

PTD was consistent across IDDSI levels, with no significant changes. Values ranged from 74.2 ± 2.18 ms at IDDSI level 2 to 80.4 ± 4.01 ms at IDDSI level 4.

Among the studied thickening powders, PT had the highest PTD at IDDSI level 3 (82 ± 23 ms), but no significant differences were observed between agents.

#### The Bolus Aspect Ratio (BAR) at the UES Opening

BAR demonstrated no significant variation across IDDSI levels or between thickening agents. The values ranged from 3.75 ± 0.18 at IDDSI level 1 to 3.57 ± 0.28 at IDDSI level 4. The QT sample had the highest BAR at IDDSI level 1 (3.99 ± 0.91), but this was not statistically significant.

## Discussion

This study evaluates the influence of gum- and starch-based thickening agents on swallowing function, with emphasis on their effects on bolus flow behaviour and extensional rheology during the transition from the oral cavity to the pharynx. A previous study, using VFSS in healthy adults, demonstarted that extensional viscosity exerts a stronger influence than shear viscosity on bolus shape at the upper oesophageal sphincter, although conclusions were constrained by analysis of only pharyngeal transit time and bolus aspect ratio, limiting complete physiological interpretation [17]. The present study extends prior research by y characterising swallowing kinematics and bolus–flow responses across five commercial thickeners – TUC (Resource Thicken Up Clear), QT (Quik Thik), SP (Supercol), PT (Purathick), and TU (Thicken Up) – formulated with barium at IDDSI levels 1 to 4 [9]. We previously reported that barium sulfate, used as a radiopaque contrast medium, can substantially modify rheological behaviour relative to non-barium controls [22]. Across all formulations, barium-containing samples exhibited higher shear viscosity at the characteristic oral processing shear rate (50 s⁻¹) than their non-barium counterparts. For gum-based thickeneres, these increases were consistent and could be described by a constant shift factor (1.21–1.73). In contrast, modified starch-based formulations exhibited inconsistent shear responses, lacking such predictive adjustments.

In terms of extensional rheology, xanthan gum-based systems exhibited reduced values in the presence of barium, suggesting diminished bolus cohesiveness, whereas guar and tara gum-based systems were largely unaffected. In contrast, starch-based systems exhibited inherently poor extensional properties, regardless of the barium content. Comparing different formulations, xanthan gum-based still demonstrated superior extensional rheological performance compared with other thickening agents. Gum-based barium-thickened liquids generally preserved IDDSI flow classification relative to matched non-barium controls, while starch-based systems exhibited disproportionately high flow resistance, achieving thicker IDDSI classifications at lower powder concentrations [22]. These results underscore that, without direct assessment of the extensional and rate-dependent shear properties of barium-thickened fluids, correlation to VFSS kinematics may remain incomplete or potentially misleading.

It is worth pointing out that all rheological measurements were conducted with the exclusion saliva effects. This limitation is particularly relevant for starch-based thickeners, which are prone to enzymatic interaction with salivary α-amylase, potentially decreasing liquid consistency during swallowing [26, 28, 29]. Future studies are warranted to examine saliva-induced consistency changes in both barium- and water-thickened liquids, especially for modified-starch-based thickeners, which are expected to be more strongly affected by salivary amylase than gum-based systems.

For VFSS evaluation, it was observed that increasing IDDSI levels were associated with a consistently higher bolus hear position within the oropharyngeal tract. Specifically, at IDDSI levels 3 and 4, the median bolus position was located in the oral cavity. This position change is clinically relevant because a bolus retained at a higher level – such as within the oral cavity – has a lower risk of premature spillage into the pharyngeal space, thereby reducing the likelihood of pre-swallow aspiration before the swallowing reflex is [30, 31]. Therefore, variations in liquid thickness play a crucial role in preventing aspiration by minimising bolus fragmentation during swallowing [6, 14, 32–34]. In healthy adults, these findings suggest that increasing viscosity may support safer swallowing by maintaining the bolus in a higher position, thereby facilitating more effective swallowing. While such an effect may also be beneficial for individuals with dysphagia, this cannot be confirmed from the present study and requires further investigation.

The relevance of this shift in bolus positioning is further supported by the results of laryngeal closure duration (LCD) and hyolaryngeal complex movement. As liquid viscosity increased, the mean LCD decreased, with a significant reduction observed for IDDSI levels 3 and 4, compared with IDDSI level 1. Prolonged laryngeal closure duration generally enhances airway protection, thereby lowering the risk of aspiration during swallowing. The higher liquid thickness, particularly at IDDSI level 4, facilitated better airway protection by maintaining a higher bolus position and delaying bolus movement into the pharyngeal cavity. This delay afforded the hyolaryngeal complex more time to elevate and protect the airway [6, 29, 35, 36].

In addition to the general effects of thicker liquids, this study also highlighted differences between gum-based and starch-based thickeners that may influence bolus movement. Gum-based agents, such as TUC and PT, tended to produce more cohesive boluses with better control over bolus flow. By contrast, starch-based agents were less stable and exhibited greater variability in their flow properties—a characteristic that may have influenced bolus transit and measures of airway protection.

The relevance of this shift in bolus positioning was supported by the results for laryngeal closure duration (LCD) and hyolaryngeal movement. As the viscosity of the thickened liquid increased, mean LCD became shorter, with a significant reduction at IDDSI levels 3 and 4 compared with level 1. While this may suggest improved airway protection, the clinical significance should be interpreted with caution, particularly as the liquids were prepared with barium sulfate, which is known to alter rheology.

This effect of thickener type was reflected in the consistently higher LCD values observed with TUC, suggesting that the bolus remained cohesive and that the hyolaryngeal complex could sustain a more protective swallowing action [11–12,37–39]. These differences were especially evident at IDDSI levels 1 and 3, where significant variations in LCD were seen between thickeners, with TUC consistently producing longer closure times. Such findings point to a potential advantage of gum-based thickeners in delaying bolus transit and providing more time for airway protection.

On the contrary, starch-based agents, i.e., TU samples, demonstrated less consistent effects on LCD and bolus position. Their greater variability in flow behaviour sometimes led to quicker bolus transit and reduced time for protective mechanisms. The lower LCD values observed for TU samples at IDDSI levels 1 and 2 highlight the challenges of maintaining bolus cohesiveness, with a higher risk of premature spillage into the pharynx and therefore aspiration. These findings are consistent with earlier studies, which have shown that gum-based products, by producing more cohesive boluses, may better support swallowing safety [11, 29, 37].

Although the values of Pharyngeal Transit Duration (PTD) and Upper Hypopharyngeal Transit Time (UHT) remained relatively unchanged across IDDSI levels, the influence of thickening agent type should not be overlooked. In contrast to the present findings, previous studies have reported a tendency for pharyngeal transit time to increase when liquids are thickened to higher IDDSI consistencies. Moreover, bolus aspect ratio (BAR) appeared to decrease with increasing IDDSI levels. Overall, these results suggest that gum-based agents promote more controlled and cohesive bolus transit, which may help reduce pharyngeal residue and improve swallowing efficiency—effects that may be particularly beneficial for individuals with impaired swallowing function [35, 40]. These findings align with earlier work showing that gum-based thickeners support bolus cohesion more effectively than starch-based formulations and may promote safer swallowing, while further emphasising that extensional rheology offers mechanistic insight that may not always be directly observable in VFSS outcomes when barium or broader physiological variability is involved [17].

Beyond the exclusion of saliva effects in the rheological and IDDSI flow measurements, this study is subject to several important limitations. Firstly, all liquids were prepared with barium sulfate to facilitate VFSS analysis; however, direct cross-comparisons of each thickening agent, both with and without barium, against IDDSI standards were not performed. Since barium alters rheological properties as described earlier, this may have influenced the swallowing outcomes. Although, matching the viscous flow curves between hydrocolloid-only and barium sulfate-containing formulations – potentially by reducing thickener concentration in the barium systems – would be a more rigorous approach for isolating thickener structural effects under controlled viscous conditions, matching the entire flow curve is technically challenging because the viscosity-increasing effect of barium is not uniform across the shear-rate range (Figure S1, Supplementary). Therefore, matching viscosity at a representative single shear rate that reflects swallowing conditions, i.e., 50 s⁻¹, may be more feasible. While this viscosity matching could improve comparability of the unmixed in-vitro samples, we acknowledge that it does not fully resolve the inherent limitation that in-vivo bolus viscosity, after mixing with saliva and exposure to salivary enzymes, will still differ from unmixed instrumental measurements. Secondly, the effect of standing time was not examined in this study, although it is particularly relevant for starch-based thickeners, which are known to exhibit time-dependent changes in viscosity. Thirdly, the study was conducted in a relatively small sample of healthy adults, and the findings cannot be generalised to individuals with dysphagia, whose swallowing physiology may differ substantially. Finally, other factors such as bolus volume, age, BMI, and underlying medical conditions were not systematically assessed. Future studies should therefore include larger and more diverse populations, investigate both barium and non-barium preparations, and evaluate clinically meaningful outcomes such as aspiration and quality of life.

## Conclusion

This study demonstrates that increasing liquid viscosity to higher IDDSI levels, particularly levels 3 and 4, promotes safer swallowing behaviour in healthy adults by maintaining the bolus in a higher oropharyngeal position and altering laryngeal closure dynamics. Gum-based thickeners consistently produced more cohesive and stable boluses with longer laryngeal closure durations, whereas the starch-based thickener exhibited greater variability in flow behaviour and reduced cohesiveness. Although key pharyngeal transit measures remained largely unchanged across viscosity levels and thickening agents, the combined rheological and VFSS findings indicate that gum-based formulations offer more reliable bolus control than starch-based counterparts when mixed with barium. Given that barium significantly modifies the rheological properties of all thickened liquids, these results should be interpreted with caution, and further research – including non-barium preparations and dysphagic populations – is required to determine the translational and clinical relevance of these findings.

## Supplementary Information

Below is the link to the electronic supplementary material.

Supplementary Material 1

## Data Availability

The datasets in the current study are not publicly available due to ethical restrictions and participant privacy concerns, particularly regarding videofluoroscopic swallowing recordings. However, data supporting the findings of this study are available from the corresponding author upon reasonable request.
