# Supplementary material for: Flow Characteristics of Barium Thickened Liquids during Swallowing in Healthy Adults
Source: Dysphagia. 2026 Feb 21;41(4):844–54. doi: 10.1007/s00455-026-10937-4 (PMC13457252; doi:10.1007/s00455-026-10937-4)
Supplement: Supplementary file 1 [file 455_2026_10937_MOESM1_ESM.docx]

**Supplementary**

|  |  |
| --- | --- |
|  |  |

**Figure S1** Shear viscosity as a function of shear rate for barium and water-thickened liquids prepared with different commercial thickening powders at concentrations corresponding to each IDDSI liquid consistency (a) IDDSI level 1, (b) IDDSI level 2 and (c) IDDSI level 3. Note that the comparsion between barium- and water-thickenend liquids was not made for IDDSI Level 4 samples across all formulations.

**Figure S2** Particle size distribution of Thicken Up (TU) sample, indicating a volume-weighted mean diameter (D [4, 3]) of 45.77 ± 0.83 μm.
